# Bridging the gap: beyond "overreach" in ECPR rewarming for hypothermic cardiac arrest in older patients

**DOI:** 10.1186/s13049-026-01580-x

**Published:** 2026-03-19

**Authors:** Tatsunori Nagamura, Yuya Yoshimura, Sho Terashige, Tetsuro Kiyozumi

**Affiliations:** 1 Department of Traumatology and Critical Care Medicine, National Defense Medical College Hospital, 3-2 Namiki, Tokorozawa, Saitama 359-8513 Japan; 2 Department of Emergency and Critical Care Medicine, Hachinohe City Hospital, Aomori, Japan; 3 Department of Orthopedics, Hamawaki Orthopaedic Hospital, Hiroshima, Japan

Dear Editor,

We sincerely thank Schöchl et al. for their valuable comments on our case report [1, 2]. Their comments highlighted important ethical, physiological, and practical considerations regarding the application of extracorporeal cardiopulmonary resuscitation (ECPR) in older patients. We welcome the opportunity to contribute to this discussion and emphasise the following three key points.

First, this report highlights the complexity of ECPR decision-making in patients with hypothermic cardiac arrest (HCA), which is not unique to older adults. Our patient had age-appropriate cognitive decline but was previously independent and had no evidence of irreversible circulatory failure. The Hypothermia Outcome Prediction after Extracorporeal Life Support (HOPE) estimated survival probability was 76%, suggesting a favourable chance of survival. Following shared decision-making principles [3, 4], we discussed ECPR and comfort care with the patient’s son as a surrogate and proceeded after careful consideration by the medical team. ECPR initiation was not immediate and required time for careful team consideration and shared decision-making with the family. These considerations were incorporated into bedside decision-making and were neither overlooked nor minimised. Although our patient required wheelchair assistance after discharge, she maintained meaningful interactions with her family and lived for an additional 4 years. Her family expressed deep gratitude for the care and viewed her survival and subsequent years as deeply meaningful. These perspectives indicate that therapeutic goals aligned with the patient and family values. In older patients, meaningful outcomes may not be fully captured by cerebral performance category (CPC) or by independent living alone. We agree that patient-reported outcome measures (PROMs) are important for evaluating ECPR outcomes. However, their application in older patients with age-appropriate cognitive decline remains challenging, as difficulties in understanding and responding to PROM instruments have been reported in this population [5, 6]. Overall, this case underscores the difficulty in prognostication and patient-centred outcome assessment in this population.

Second, justice and resource allocation cannot be defined by a single universal standard, either international or medical. In our case, the intensive care unit (ICU) capacity was sufficient, and the initiation of ECPR did not limit treatment opportunities for other patients. The appropriateness of ECPR should be interpreted within local healthcare systems and patient-centred values, particularly in aging societies, where resource considerations are increasingly important. Patient- and family centred outcome frameworks, including PROMs, will become vital in future debates on ECPR for older patients. Our report does not support routine ECPR in all older patients, but suggests that meaningful survival in carefully selected older patients may have important implications for decision-making and resource allocation.

Third, regarding medical indications, HCA represents a potentially reversible condition that requires decision-making distinct from normothermic cardiac arrest. The 2025 Extracorporeal Life Support Organization (ELSO) narrative guideline states that age ≥70 years is not a contraindication for ECPR rewarming in HCA [7]. Moreover, successful cases of ECPR rewarming with favourable neurological outcomes have been reported in nonagenarians [8, 9], highlighting the need for an individualised assessment beyond age. Although the HOPE score does not fully account for frailty or age-specific comorbidities, we do not consider it a standalone determinant but rather a pragmatic tool to support clinical judgement. Our report supports its clinical relevance, even in patients of advanced age, when interpreted comprehensively.

In conclusion, this report highlights the difficulty of prognostication and decision-making in ECPR and the importance of careful physiological and ethical evaluations. The appropriateness of initiating ECPR cannot be determined by a single universal standard but should be interpreted within the local healthcare systems, available resources, and patient-centred values. HCA is a potentially reversible condition, and ECPR rewarming decisions should not be based solely on age. When ethical and physiologic principles are met, ECPR rewarming for HCA in older patients is not necessarily “overreach” but rather can offer “HOPE” to patients and their families.

## Data Availability

No datasets were generated.

## Abbreviations

ECPR: Extracorporeal cardiopulmonary resuscitation
HCA: Hypothermic cardiac arrest
HOPE: Hypothermia Outcome Prediction after Extracorporeal Life Support
CPC: Cerebral Performance Category
PROMs: Patient-reported outcome measures
ICU: Intensive care unit
ELSO: Extracorporeal Life Support Organization
